# Real Time Corner Detection for Miniaturized Electro-Optical Sensors Onboard Small Unmanned Aerial Systems

**DOI:** 10.3390/s120100863

**Published:** 2012-01-12

**Authors:** Lidia Forlenza, Patrick Carton, Domenico Accardo, Giancarmine Fasano, Antonio Moccia

**Affiliations:** 1 Department of Aerospace Engineering (DIAS), University of Naples “Federico II”, P.le Tecchio 80, Naples I80125, Italy; E-Mails: lidia.forlenza@unina.it (L.F.); g.fasano@unina.it (G.F.); antonio.moccia@unina.it (A.M.); 2 Department of Systems Control and Flight Dynamics (DCSD), ONERA, Toulouse 31055, France; E-Mail: Patrick.Carton@onera.fr

**Keywords:** corner detection, efficiency, Harris/Shi-Tomasi algorithm, airborne unmanned platforms

## Abstract

This paper describes the target detection algorithm for the image processor of a vision-based system that is installed onboard an unmanned helicopter. It has been developed in the framework of a project of the French national aerospace research center Office National d’Etudes et de Recherches Aérospatiales (ONERA) which aims at developing an air-to-ground target tracking mission in an unknown urban environment. In particular, the image processor must detect targets and estimate ground motion in proximity of the detected target position. Concerning the target detection function, the analysis has dealt with realizing a corner detection algorithm and selecting the best choices in terms of edge detection methods, filtering size and type and the more suitable criterion of detection of the points of interest in order to obtain a very fast algorithm which fulfills the computation load requirements. The compared criteria are the Harris-Stephen and the Shi-Tomasi, ones, which are the most widely used in literature among those based on intensity. Experimental results which illustrate the performance of the developed algorithm and demonstrate that the detection time is fully compliant with the requirements of the real-time system are discussed.

## 1. Introduction

Unmanned Aerial Systems (UAS) can be devoted either to military or to civil missions, such as monitoring of boundaries, crop surveying, and search and rescue at disaster sites [1–6]. For this reason, there has been a remarkable flurry of research concerned with increasing the level of autonomy of UAS, thus allowing them to perform fully autonomous vision-based take-off and landing, and collision avoidance [7–9]. In this framework, the ONERA French national aerospace research center carries out the ReSSAC (Search and Rescue by Cooperating Autonomous System) project [10]. The main objective of this project is to implement a vision-based navigation and target tracking system onboard an unmanned helicopter that has to fly in an unknown urban environment.

**Figure 1.**
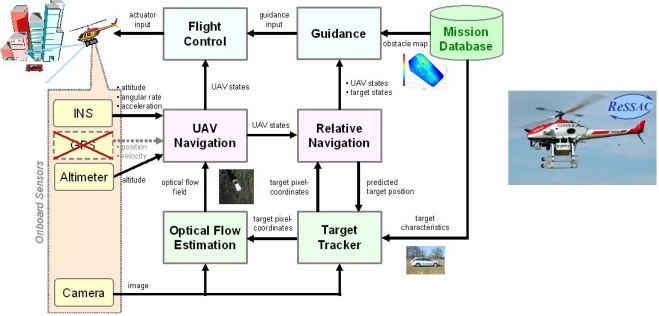
Vision based air-to-ground target tracking system architecture.

Figure 1 illustrates the flying experimental platform and the onboard system architecture of the vision-based air-to-ground target tracking system. It is worth noting that three functions must be carried out by the image processor: target detection, target tracking, and ground motion estimation by means of optical flow.

This paper describes a customized target detection technique which is based on the corner detection method. Currently, corner detection is widely used in many applications such as object identification [11] and tracking in real-time systems [12–14]. However, it is demanding in terms of computational effort. For this reason, we have developed a computationally light version of a corner detection algorithm based on image intensity; in particular, it exploits the Harris-Stephen and Shi-Tomasi criteria [15,16], which highlight the image details with high accuracy. The algorithm has been coded in Visual DSP C++ language, for a dual-core symmetric multiprocessor. The advantage of choosing that particular processor configuration is that it can run different tasks on each of the cores at the same time, allowing the processing unit to save a great deal of computation time. Moreover the overall electro-optical system is suitable for unmanned platforms such as micro-UAVs, thanks to its compactness and lightness. The final part of the paper focuses on algorithm performance, demonstrating that it is suitable for a real time system, where short computation time is mandatory [17–19]. Besides the processing time, the innovation brought by our work consists in customizing the algorithm implementation for a miniaturized smart sensor that can be used also in very small unmanned platforms such as microdrones.

## 2. Requirements and Project Background

The ReSSAC project is set within a scientific framework whose research goal is to increase the decisional autonomy of Unmanned Aerial Vehicles (UAVs). This concept would bring many advantages to the civil as well as the military fields; indeed drones are an important complementary technology to remote sensing by satellites, they can provide search and rescue support, or target/vehicle tracking, and, moreover, they allow creating cooperative sensor networks which operate in wide operating fields.

As regards the ReSSAC project, it aims at developing a fully autonomous helicopter able to perform:

- Guidance, Navigation and Control;
- Data collecting and processing;
- Landing maneuvers on unknown areas.

The flying experimental platform (Figure 1) is the Yamaha R-MAX, already used by several research centres, such as NASA, Linkoping University, Carnegie-Mellon University, UC Berkeley [20–23]. As regards its technical characteristics, it weighs 60 kg, has a length of 3.63 m and carries up to 20 kg payload.

The ability of performing autonomous landings at unprepared sites is a crucial security and efficiency issue, and terrain characterization is a necessary step for UAVs, when they autonomously select a landing location. The ONERA ReSSAC helicopter studies the terrain by means of a nadir-mounted camera, which applies a monocular stereovision technique, based on the motion of the UAV. In particular stereovision algorithm roughly works in the following way:

1. Selection of points of interest in the image;
2. Matching of the selected points between two following images;
3. Triangulation and estimation of the relative localization of objects corresponding to these points.

**Figure 2.**
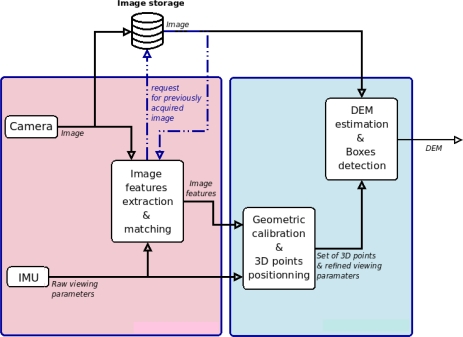
General architecture of terrain characterization from image sequence.

Figure 2 shows how the three modules composing the image processing chain are organized to map the terrain from an image sequence.

This paper is focused on the algorithm realized for implementing the first point which regards the automatic point extraction, performed on each image. It has been improving since its first version, which was very heavy and impossible to use in a real-time system [24]. This paper describes the last algorithm version that results the fastest and lightest; in fact it is able to look for 100 points of interest in less than 60 ms.

## 3. Criterions of Detection of the Points of Interest

Image features, or points of interest are a very broad concept which, generally, indicates the image points with particular characteristics, used to match two or more consecutive images. From the Harris point of view [16], an image feature is a corner, detected by computing on each pixel a saliency degree taking into account the local texture surrounding the considered pixel. Texture is related to local variations of pixel’s intensity around the considered point. In particular, the corner detection criterion is based on a score calculated for each pixel from two eigenvalues of the image, considered as matrix; after that, the searching of score maximum values is implemented; they correspond to the image corners.

The Shi-Tomasi corner detector is based entirely on the Harris corner detector [15]. However, this method differs from the previous one in the pixel score evaluation, which depends only on eigenvalues, in order to determine if a pixel is corner or not. In detail, we illustrate the equations that characterize the two methods and that provide more clearly their differences.

Let us consider the image array *I*(*x*,*y*), with x and y respectively horizontal and vertical pixel indexes, and let us define *I_x_*(*x*,*y*) and *I_y_*(*x*,*y*) the first order directional differentials, provided by a differential operator, such as Sobel, Prewitt, Roberts *etc*. [25]. We can build the symmetric autocorrelation matrix S in the neighborhood of the pixel (*x*,*y*) in the following way:

(1) $$S(x,y)=\sum_{\varsigma ,\eta }w(\varsigma ,\eta )\left[\begin{array}{cc} I_{x}^{2}(x+\varsigma ,\text{y}+\eta ) & I_{x}(x+\varsigma ,y+\eta )I_{y}(x+\varsigma ,y+\mu )\\ I_{x}(x+\varsigma ,y+\eta )I_{y}(x+\varsigma ,y+\eta ) & I_{y}^{2}(x+\varsigma ,y+\eta ) \end{array}\right]$$

where *w*(*ξ*,*η*) is a smoothing function that weights differently the points of the considered neighborhood; its characteristic function can be square, triangular or Gaussian. For more detail, the description of the principle of choice of the type of smoothing filter for our application is postponed to later sections.

Let us observe that the obtained matrix *S* is positive semidefinite, so that it has the important properties that all the eigenvalues are real and nonnegative [26]. Let us indicate those eigenvalues with *λ*_1_ and *λ*_2_, given by of the second order equation:

(2) $$\lambda^{2}-\lambda \;\times \;\text{track}(S)+\text{det}(S)=0$$

Both Harris and Shi-Tomasi methods are based on pixel scores, depending on eigenvalues.

Indeed, Harris calculates that score as explained hereinafter:

(3) $$C_{\text{Harris}}\;(x,\;y)=\text{det}[S(x,\;y)]-{k*\text{track}}^{2}\;[S(x,\;y)]$$

where *k* is an empirical value, usually fixed as 0.06 [27], and det[*S*(*x*,*y*)] and track[*S*(*x*,*y*)] depend on the eigenvalues by the following equation:

(4) $$\det[S(x,y)]=\lambda_{1}\lambda_{2}$$

(5) $$\mathrm{track}[S(x,y)]=\lambda_{1}+\lambda_{2}$$

On the other hand, the Shi-Tomasi method evaluates the pixel score on the basis of a simpler relation Equation (6):

(6) $$C_{\text{Tomasi}}\;(x,y)=\text{min}\left(\lambda_{1},\lambda_{2}\right)$$

Maximum values of *C*(*x*,*y*) parameter are the image points of interest, both in the Harris and in the Shi-Tomasi cases. Therefore, when the user asks for a selected number of corners, the algorithm lists the *C*(*x*,*y*) values in ascending order, and provides the position of pixels which correspond to the first values of the list, on the basis of the requested number of corners.

## 4. Laboratory Test System Architecture

**Figure 3.**
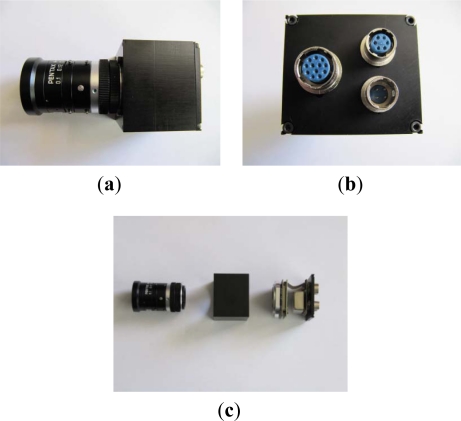
Drone EO camera layout: (**a**) intelligent camera; (**b**) external camera connections; (**c**) main camera components.

The image processing algorithm configuration and testing have been executed in the laboratory, verifying the results on a spare monochromatic camera which is the same of the onboard Electro-Optical (EO) system of the drone. Figure 3 shows the camera in detail. It has reduced weight and size (43 × 38 × 38 mm) and it is characterized by three external connections which provide:

- the communication with the programming computer by means of a SPI bridge (SC18IS600 model, manufactured by NXP), in order to implement and to test the image processing algorithms;
- the link with the host computer by means of a RS422 serial bus;
- the power connection.

Moreover the complementary metal oxide semiconductor (CMOS) image sensor is manufactured by Micron (model MT9P031). Its active surface is of 5.7 × 4.28 mm and the pixel size is of 2.2 × 2.2 μm. The focal length is variable from 6 mm to 16 mm as well as the resolution, which can be reduced from 2,592 × 1,944 pixels to 1,296 × 972 pixels or 648 × 486 pixels by means of binning or subsampling operations. The camera data rate is 14 Hz at full resolution and it increases at 123 Hz in Video Graphics Array (VGA) resolution (640 × 480). Moreover, camera is characterized by a quantum efficiency of 27% in the visible spectral range (390–750 nm), while optics has a type S mounting, M12 × 0.5 thread.

**Figure 4.**
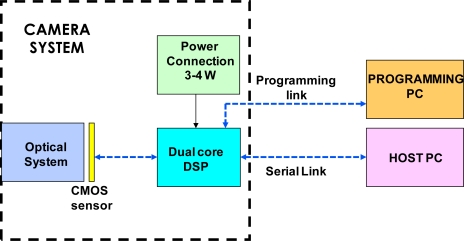
Laboratory test system.

Indeed, the electronic camera system is composed by two more electronic boards, such as a processor and a Power Control Unit (PCU). The particular processor (Blackfin family, model ADSP BF561) is manufactured by Analog Device [28]. It is composed by dual symmetric 600 MHz high performance cores and 328 K of total on-chip memory (L1 memory). Moreover the processor has a more internal L2 memory of 128 K and Direct Memory Access (DMA) controllers which provide the access to the 120 MHz Static Random Access Memory (SRAM) off-chip memory of 64 MB (L3 memory). That ADSP system is connected to two computers: one is dedicated to the algorithm development; the second is the host computer which communicates with the ADSP by means of the serial link and controls the algorithm execution and configuration (sensor exposition time, acquisition duration). Figure 4 illustrates the laboratory test system scheme.

The selected hardware has been customized for our applications, in order to satisfy requirements for real-time system onboard unmanned platforms, and also for microUAV’s. Thus, all the electro-optical system development has been adapted to the choice of the processor, which was demanded to be dual core. Subsequently, camera black box and PC to ADSP connections have been selected and realized. Thus, a low-level programming tool has needed to have direct access to the processor, in order to implement the desired functions.

## 5. Improved Corner Detection Algorithm

**Figure 5.**
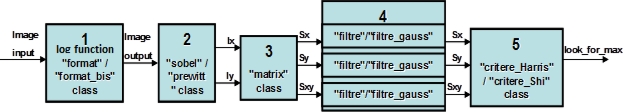
Corner detection algorithm blocks diagram.

The corner detection algorithm has been developed in Visual DSP C++ language. Its main purpose is to reduce the computation time at less than 60 ms, also at large image resolution (VGA mode). Figure 5 illustrates the blocks diagram of the implemented algorithm. Thus, the reader can observe that the algorithm consists of six blocks, each one of which has a specific function and corresponds to a C++ class. Classes’ characteristic names are: “format”/“format_bis”, “sobel”/“prewitt”, “matrix”, “filtre”, “critere_Harris”/“critere_Shi”. Hereinafter each function is described in detail.

### 5.1. “Format”/“Format_bis” Class: Selection of Image Output Format

The first operation executed is the image copying from the off-chip to the on-chip memory. It is represented by the first block of Figure 5 and it is called by the “format” or the “format_bis” classes. In particular the latter one implements also the image binning, providing in output an image with quarter size of the input one. Both functions are very advantageous because they allow a great gain of computational time with respect to the simple image reading on the off-chip memory; in fact, in this case the processor spends only 4 cycles per 4 pixels at 120 MHz, instead of 26 cycles per 4 pixels at 120 MHz in the case of direct reading on the off-chip memory. Thus, for the sake of clarity, this class spends 76,800 cycles to read the entire VGA image, with respect to 1,996,800 cycles in the second case. The reader can observe that the “log function” is also mentioned in this block, but it will be analyzed more in detail in what follows.

### 5.2. “Sobel”/“Prewitt” Class: Implementation of Edge Detection Method

The first image treatment applied to the image, and indicated in block 2, is the edge detection, according to the Sobel or the Prewitt method [25,29] and called in the C++ script by the “sobel” and “prewitt” classes. Both of them receive in input three consecutive “format” (or “format_bis”) output rows and perform their convolution with the horizontal and vertical filters of the chosen method; from the second iteration, the first row of the previous iteration is lost and the new output from “format” is gained and added below to the other two rows already kept in memory. This is repeated until the end of the image. As regards the Sobel operators, the horizontal and vertical filters are represented by the following [3 × 3] matrices:

(7) $$F_{H}^{\text{sobel}}=\frac{1}{4}\left[\begin{array}{ccc} -1 & 0 & 1\\ -2 & 0 & 2\\ -1 & 0 & 1 \end{array}\right]=\frac{1}{4}\left[\begin{array}{c} 1\\ 2\\ 1 \end{array}\right]*\left[\begin{array}{lll} -1 & 0 & -1 \end{array}\right]$$

(8) $$F_{V}^{\text{sobel}}=\frac{1}{4}\left[\begin{array}{ccc} -1 & -2 & -1\\ 0 & 0 & 0\\ 1 & 2 & 1 \end{array}\right]=\frac{1}{4}\left[\begin{array}{c} -1\\ 2\\ 1 \end{array}\right]*\left[\begin{array}{lll} 1 & 2 & 1 \end{array}\right]$$

whereas, the Prewitt filters are:

(9) $$F_{H}^{\text{prewitt}}=\frac{1}{3}\left[\begin{array}{ccc} -1 & 0 & 1\\ -1 & 0 & 1\\ -1 & 0 & 1 \end{array}\right]=\frac{1}{3}\left[\begin{array}{c} 1\\ 1\\ 1 \end{array}\right]*\left[\begin{array}{lll} -1 & 0 & 1 \end{array}\right]$$

(10) $$F_{V}^{\text{prewitt}}=\frac{1}{3}\left[\begin{array}{ccc} -1 & -1 & -1\\ 0 & 0 & 0\\ 1 & 1 & 1 \end{array}\right]=\frac{1}{3}\left[\begin{array}{c} -1\\ 1\\ 1 \end{array}\right]*\left[\begin{array}{lll} 1 & 1 & 1 \end{array}\right]$$

### 5.3. “Matrix” Class: Building of the Structure Tensor Components

This function estimates the components of the second order moment matrix *J*(*x*,*y*) that is needed to build the tensor *S*(*x*,*y*) in Equation (1). In particular it receives the gradient function outputs, *Ix* and *Iy*, from the chosen edge detection method of block 2, and provides the diagonal and off-diagonal *J* components:

(11) $$•\;\;\;\;\text{diagonal components}:J_{11}(x,y)=I_{x}^{2},\;J_{22}(x,y)=I_{y}^{2}$$

(12) $$•\;\;\;\;\text{off-diagonal components}:J_{12}(x,y)=J_{21}(x,y)=I_{x}•I_{y}$$

### 5.4. “Filtre”/“Filter_Gauss” Class: Performing of Filtering on the Tensor Components

The fourth algorithm block implements several smoothing filters on the tensor S components, such as the square, the triangle, the Hanning and the Gaussian filters [29,30]. In this block only two class names are indicated, “filtre” and “filtre_gauss”, which correspond to the square and the Gaussian filters. In fact the triangle and the Hanning filters are implemented calling twice and three times, respectively, the square filtering. The main effect of square filtering is the removal of high spatial frequency noise by averaging the intensity of pixel on a selected window.

The square filter algorithm could appear heavy in computational terms, since it requires computation of a sum of intensities on a selected window for each pixel in the processed image area. However, a significant reduction of the computational load can be obtained by performing a recursive computation of the above mentioned sum, *i.e.*, the values of sum of pixel intensities in the in the intersection of windows must not be recomputed when the center of the window moves from a pixel to its immediate neighbor [31].

As regards, the triangle and the Hanning filters, they require twice and three times, respectively, the computation time of the square filter. As regards the Gaussian filter, its size is variable from 3 × 3 to 11 × 11. In this case the matrix coefficients are calculated on the basis of the Gaussian function:

(13) $$g(l)=e^{-\frac{l^{2}}{{2\sigma }^{2}}}$$

where l varies between −M/2 and +M/2 (M is the filter size) and *σ* is the standard deviation which we have assumed 2.2. As for the Sobel and the Prewitt filters, also the Gaussian filter can be decomposed in two simpler vectors which perform the vertical and the horizontal convolution, respectively. Therefore, the filter matrix decomposition is explained below:

(14) $$G=\left[\begin{array}{ccc} g(-M,-M) & g(0,-M) & g(M,-M)\\ g(0,-M) & g(0,0) & g(M,0)\\ g(M,-M) & g(0,M) & g(M,M) \end{array}\right]=\left[\begin{array}{c} g(-M)\\ g(0)\\ g(M) \end{array}\right]*\left[\begin{array}{ccc} g(-M) & g(0) & g(M) \end{array}\right]$$

Moreover, hereinafter we present in Table 1 the filter coefficients, for different filter sizes. Each of them has been normalized with respect to the coefficients total sum and it is multiplied for 256, which is the maximum allowed intensity:

### 5.5. “Critere_Harris”/“Critere_Shi” Class: Providing of the Criterions of Detection of Points of Interest

The fifth block applies the criteria of detection of the points of interest on the basis of the Harris-Stephen or the Shi-Tomasi methods. They are provided by the “critere_Harris” and “critere_Shi” classes of the C++ script on the basis of Equations (3) and (6).

From the computation time point of view, the Shi Tomasi criterion results in the heaviest load, in fact it requires the resolution of a square root, in order to find the solutions of the second degree Equation (2). Therefore, we have considered an approximated square root, based on a table of 256 values providing a precision of 0.2%, in order to lighten the algorithm.

### 5.6. “Look_for_Max”: Looking for Maximum Values

Firstly, it implements the search of maximums in each row of the image, on the basis of the selected criterion. In particular it provides the comparison of pixels value within the same, the previous and the following rows and it outputs the maximum detected value and the x and y coordinates of the correspondent pixel. Secondly, it executes a new search of maximums, dependent on the number of points requested by user (usually 100). Outputs from the second search correspond to the image corners or points of interest.

## 6. Algorithm Performance Analysis

**Figure 6.**
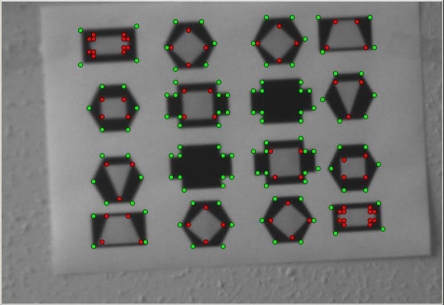
Test Image.

In order to examine our corner detection algorithm performance, we have considered Figure 6 as the test image that consists of most types of junctions (*L*, *T*, *X* and *Y*), moreover similar images have been already widely used [14,17,18] to test how an algorithm responds to different types of geometries. In particular, our reference image is characterized by 182 points of interest, 112 of which are external points (the green ones), while 70 are internals (the red ones).

Setting the image at different brightness conditions, we have observed that the points of interest of the brighter zones prevailed over the darker ones. Therefore in the first block algorithm, we have added the implementation of a logarithmic transformation which expands the values of dark pixels while compressing the higher-level values [32], on the basis of the following relation:

(15) $$I_{g}\;(x,y)=\text{log}\;[I(x,y)]$$

where *I*(*x*,*y*) represents the input pixel numerical value, while *I_g_*(*x*,*y*) is the output pixel value, after logarithmic correction. This function resulted as a good solution applicable everywhere, before the corner detection algorithm and it is indicated in the first block of the diagram in Figure 5 by “log function”.

Afterwards, we have evaluated the algorithm detection time on the basis of the number of operations per pixel for each function in the blocks of Figure 5. Table 2 synthesizes the functions characteristics; in particular each of them has been indicated according to the names used in the C++ script.

Let us observe that the square filtering computation time is independent of its filter size, and it is always constituted by 10 instructions per pixel, implemented three times, one for each image tensor component. On the other hand, Gaussian filtering is computationally heavy, because it depends on the filter size and it is constituted by 30 instructions as a minimum, multiplied by three. The triangle and the Hanning filtering are performed by applying the square filtering two or three times. Therefore the lightest filtering is the square window, which is the most suitable for our applications.

Regarding the choice of the criterion of selection of the points of interest, from our analysis the Harris method turns out the fastest one, because the Shi-Tomasi method involves the implementation of the square root, which is very computationally heavy, even if it is applied in the form of lookup table.

## 7. Results: Assessed Performance

Finally we can assess the best algorithm configuration in order to obtain the smallest computational weight. Table 3 resumes the selected functions and it reports the algorithm computation time as for the binned VGA as for the full VGA format in the case of looking for 100 points of interest.

It is worth noticing that the evaluated algorithm processing time is very close to the theoretical value. The latter is obtained as follows: the sum of the total operations number is multiplied by the image format, VGA or binned VGA, and divided by the reading frequency. In particular, for the algorithm configuration of Table 3, the theoretical time processing of a VGA image is: (640 × 480) × (10 + 14 + 8 + 30 + 10 + 6)/600000 = 39.9 ms. That is a good result because the algorithm spends about only 5 ms more than the theoretical time, to perform the external operations of calling the functions and accessing the memory. Thus, computation time results respect requirements, as for VGA as for binned VGA images.

**Figure 7.**
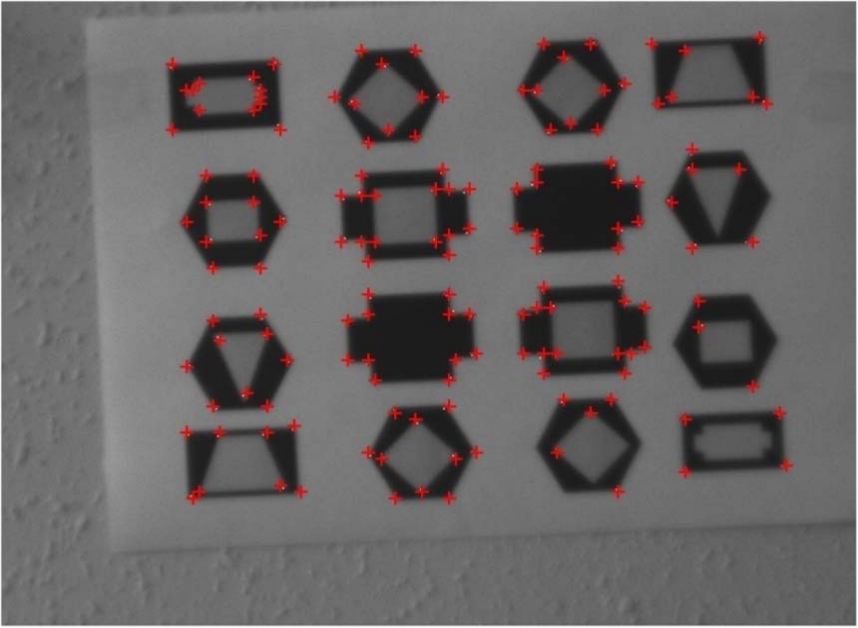
Corner detection implemented on the reference image.

Figure 7 shows an example of the algorithm implementation on the test image of Figure 6 in VGA format setting the parameters described in Table 3. In this case we have asked the algorithm to find 150 points, more than algorithm requirement, in order to make outputs more clearly visible to the reader. Indeed, detected points are indicated by red crosses; therefore the reader can observe that all of them correspond to corners of the represented geometric figures. Flight tests in real scenarios will of course introduce additional noise effects. However, vibration effects will be significantly reduced by the camera isolation structure and the attitude control system.

## 8. Conclusions

A fast corner detection algorithm has been described. It has been realized within the framework of the ReSSAC ONERA project, which aims at realizing a fully autonomous helicopter, able to detect and track targets and to estimate ground motion. The proposed technique has been studied and applied for providing the target detection function. It is based on the detection of “points of interest”. The proposed paper has presented the analysis carried out in order to evaluate the best algorithm configuration in terms of speed of execution. In particular two criteria of detection of the points of interest have been studied and compared: the Harris-Stephen and the Shi-Tomasi ones. The first one has been estimated as the lightest one from a computational point of view. In particular, considering the implementation of square window filtering, the overall computational time is very short, both in the case of full and binned VGA image formats (45 ms and 13 ms, respectively). The experimental analysis has also demonstrated a satisfying corner detection capability, with a large number of corners being detected in a reference image. Further developments, foreseen in the ReSSAC project, consist in integrating the camera on the helicopter and testing the algorithm on real images, taken during flight tests, with the aim of evaluating its performance in different light conditions and with real objects to detect.

## Figures and Tables

**Table 1.** Gaussian Filter Coefficients.

**Filter Size**	**Filter Coefficients in Symmetric Order**										
3 × 3	0	0	0	0	42	171	42	0	0	0	0
5 × 5	0	0	0	14	62	103	62	14	0	0	0
7 × 7	0	0	7	27	57	74	57	27	7	0	0
9 × 9	0	5	14	31	49	57	49	31	14	5	0
11 × 11	3	9	18	31	42	47	42	31	18	9	3

**Table 2.** Algorithm functions characteristics.

**Function Name**	**Operations Number/Pixel**
“format”	10
“format_bis”	14
“sobel”	15
“prewitt”	14
“matrix”	8
“filter”	10 × 3
“filtre_gauss”	(12 + 6 × (filter size)) × 3
“critere_Harris”	10
“critere_Shi”	31
“look_for_max”	6

**Table 3.** Corner detection algorithm configuration and performance.

**Edge Detection Method**	**Prewitt**
Filter Size and Type	(7 × 7) square filter
Criterion of Detection of Corners	Harris-Stephen
Detection Time on VGA Image	45 ms
Detection Time on Binned VGA Image	13 ms
